# UAV spraying on citrus crop: impact of tank-mix adjuvant on the contact angle and droplet distribution

**DOI:** 10.7717/peerj.13064

**Published:** 2022-03-11

**Authors:** Yanhua Meng, Wanqiang Zhong, Cunjia Liu, Jinya Su, Jiyuan Su, Yubin Lan, Zhiguo Wang, Meimei Wang

**Affiliations:** 1 School of Mechanical Engineering, Anyang Institute of Technology, Anyang, Henan Province, China; 2 Department of Aeronautical and Automotive Engineering, Loughborough University, Loughborough, Leicestershire, United Kingdom; 3 Key Laboratory of Aviation Plant Protection, Ministry of Agriculture and Rural Affairs, Anyang, China; 4 College of Electronic Engineering, South China Agricultural University, Guangzhou, Guangdong Province, China; 5 School of Computer Science and Electronic Engineering, University of Essex, Colchester, Essex, United Kingdom; 6 Guilin Jiqi Biochemical Company, Guilin, Guangxi Zhuang Autonomous Region, China

**Keywords:** Unmanned aerial vehicle (UAV), Tank-mix adjuvant, Contact angle, Citrus, Droplet distribution

## Abstract

Adding tank-mix adjuvants into the spray mixture is a common practice to improve droplet distribution for field crops (*e.g*., rice, wheat, corn, *etc*.) when using Unmanned Aerial Vehicle (UAV) sprayers. However, the effectiveness of tank-mix adjuvant for UAV spraying in orchard crops is still an open problem, considering their special canopy structure and leaf features. This study aims to evaluate the effects of a typical tank-mix adjuvant concentrations (*i.e*., Nong Jian Fei (NJF)) on Contact Angle (CA) and droplet distribution in the citrus tree canopy. Three commonly used parameters, namely dynamic CA, droplet coverage, and Volume Median Diameter (VMD), are adopted for performance evaluation. The dynamic CAs on the adaxial surface of citrus leaves, for water-only and NJF-presence sprays, respectively, are measured with five concentration levels, where three replications are performed for each concentration. The sprays with 0.5‰ NJF are adopted in the field experiment for evaluating droplet distributions, where Water Sensitive Papers (WSPs) are used as collectors. Two multi-rotor UAVs (DJI T20 and T30) which consist of different sizes of pesticide tanks and rotor diameters are used as the spraying platforms. Both water-only and NJF-presence treatments are conducted for the two UAVs, respectively. The results of the CA experiment show that NJF addition can significantly reduce the CAs of the sprays. The sprays with 0.5‰ NJF obtain the lowest CA within the observing time, suggesting a better spread ability on solid surface (*e.g*., WSPs or/and leaves). With respect to the effects of NJF addition on individual UAVs, the field trial results indicate that NJF addition can remarkably increase both the droplet coverage and VMD at three canopy layers, except for T30 droplet coverage of the inside and bottom layers. Comparing the difference of droplet coverage between two UAVs, while significant difference is found in the same layer before NJF addition, there is no notable difference appearing in the outside and bottom layers after NJF addition. The difference of VMD in the same layer between two UAVs is not affected by NJF addition except for the bottom layer. These results imply that the differences of droplet coverage among different UAVs might be mitigated, thus the droplet distribution of some UAVs could be improved by adding a tank-mix adjuvant into the sprays. This hypothesis is verified by investigating the droplet penetration and the correlation coefficient (CC) of droplet coverage and VMD. After NJF addition, the total percentage of T20 droplet coverage in the bottom and inside layers is increased by 5%. For both UAVs, the CCs indicate that both droplet coverage and VMD increase at the same time in most cases after NJF addition. In conclusion, the addition of a tank-mix adjuvant with the ability to reduce CA of the sprays, can effectively improve droplet distribution using UAV spraying in the citrus canopy by increasing droplet coverage and VMD.

## Introduction

With the rapid growth of global population, the overall requirements of agricultural products from human beings have been continuously increasing (Dhananjayan, Jayakumar & Ravichandran, 2020). According to the prediction of the Food and Agriculture Organization of the United Nations (FAO), the world population is expected to reach 9 billion by 2050, and the need for agricultural products will increase by 70% (Sylvester, 2018). Coupled with human’s increasingly high demand for the quality of life and the requirement of a sustainable use of resources, strategies for crop pests and diseases management are becoming even critical. The application of pesticides during crop cultivation is the main way to avoid yield loss and ensure the quality of agricultural products due to pests and diseases (Dhananjayan, Jayakumar & Ravichandran, 2020; Kalia & Gosal, 2011). However, agrochemical spray droplets depositing in the off-target areas would cause damage(s) to non-target receptors, such as air, water, animals, and plants, *etc*. (Felsot et al., 2010; Melo et al., 2015). How to reduce the notable damage caused by pesticide application to human health, environment, and ecosystems has attracted much attention in the last few decades (Mahmud et al., 2021). In particular, the overuse and inappropriate use of pesticides is becoming a rising concern. As a critical element of precision agriculture, precise pesticide application is a suitable adoption for a sustainable agricultural development. Drones, also known as Unmanned Aerial Vehicles (UAVs) or Unmanned Aerial Systems (UASs), have become a new sprayer for crop management (*e.g*., pests and diseases) in many countries since the last decade, which is mainly due to their high efficiency, flexibility, and mobility in variable meteorological and terrain conditions (Barbedo, 2019; Giles, 2016; He, 2018; Li et al., 2020; Radoglou-Grammatikis et al., 2020).

As one of the leading fruit crops globally, citrus is widely cultivated worldwide due to its high demand from human diets (Lado, Rodrigo & Zacarías, 2014; Liu, Heying & Tanumihardjo, 2012). During the cultivation process, citrus crops usually face harm from various pests and diseases, which result in a yield reduction and fruit quality depreciation (Dhananjayan, Jayakumar & Ravichandran, 2020; Tennant et al., 2009). Pesticides are required for the control of insects, pests, diseases, and weeds for the consistent production of tree fruits (Mahmud et al., 2021). In practice, pesticides sprayed continuously by conventional ground-based sprayers in orchards could be wasted significantly because of the considerable spacing between rows, which also results in environmental pollution (Asaei, Jafari & Loghavi, 2019). Furthermore, ground-based sprayers for pesticide application are subject to terrain restrictions and labor-consuming in small or mountainous and hilly orchards (Wang et al., 2019). Therefore, in the main fruit-producing areas, UAVs are gradually developed as a new sprayer to overcome labor shortages, geographic restrictions, and short-term window periods for pests and diseases control (Meng et al., 2020; Zhang et al., 2016, 2017). Recently, studies on UAV application in orchards are mainly focused on disease monitoring and pesticide or nutrient spraying during pests and diseases management processes (Martinez-Guanter et al., 2019; Tang et al., 2018; Zhang et al., 2017). For pesticide application, the optimization of spraying parameters, such as flight height, velocity, nozzle flow rate, and application rate, is explored in some existing studies (Li et al., 2020; Meng et al., 2020; Zhang et al., 2021). Some previous studies have investigated different UAV operational parameters for pesticide application to obtain an ideal control efficacy in citrus orchards. Hou et al. (2019) investigates the effects of flight height and velocity on droplet distribution in citrus trees, and the results show that flight velocity contributes to 74% of the effect on droplet deposition. Zhang et al. (2016) and Tang et al. (2018) have studied the effects of UAV operation height and tree shape on droplet deposition in citrus trees, and the results indicate that both have significant effects on droplet distribution in the tree canopy. Other previous research has also shown that the control efficacy of certain pests and diseases in citrus orchard is acceptable (Zhang et al., 2017).

Considering spraying characteristics, in addition to the difference of application rate between ground- and UAV-based application, droplets of UAV spraying need to travel a longer distance before depositing on target crops, which might increase droplet drift potential (Wang et al., 2020). Hence, it is an essential thing to improve droplet distribution in the target area when UAVs are employed as sprayers. For the process of pesticides applied by spraying, spraying quality of sprayers, the property of spraying liquids, and the spraying technologies are the three mostly considered aspects (Ahmad et al., 2021). Droplet distribution of UAV spraying can be improved by a variety of methods, such as using the optimal spraying parameters to get a satisfactory droplet coverage and penetration, adjusting droplet size to reduce droplet drift, and altering the physicochemical properties of sprays to increase droplet deposition. The research perspectives of the studies related to UAV spraying mentioned above are mainly from the angles of sprayers and spraying technologies, while the influences of spraying liquid property on droplet distribution are rarely noticed. Contact angle (CA), which quantifies the wettability of a solid surface by a liquid, is the geometric angle made between a solid/liquid/gas interface on a surface, and it is an important metric for evaluating the likeness of a liquid on wetting the solid surface (Dwivedi et al., 2017; Gribanova, Kuchek & Larionov, 2016; Quetzeri-Santiago, Castrejón-Pita & Castrejón, 2020). Low CA values indicate that the liquid could spread and adhere to the solid surface easily, whereas large CA values indicate the difficulty of a liquid to wet the solid surface due to repelling of solid surface to water (Huhtamki et al., 2018). Thus, for evaluating the wettability of the leaf surface or the effects of adjuvants on droplet spreading ability, the metric CA of spraying liquid on the leaf surface are usually adopted (He et al., 2019; Li et al., 2019). The CA of water on the surface of a hydrophobic leaf is large, which prevents water from spreading and makes the water droplets slide off leaf surface easily. However, in practice, most pesticides are diluted in water for spraying. Therefore, reducing the CA of the spraying liquids to facilitate them spreading on leaf surface is an important way to reduce off-target deposit (Melo et al., 2015). Most tank-mix adjuvants aim for reducing evaporation, spray drift, and volatilization and improving pesticide’s ability to spread and stick on the target surface by reducing CA (Celen, 2010; Melo et al., 2015; Santos, Ferreira & Viana, 2019). The addition of tank-mix adjuvant(s) into a pesticide product or pesticide spray mixture contributes to the reduction of spraying droplets’ CAs, which is expected to reduce off-target deposit effectively and obtain a better distribution (Griesang et al., 2017; Mehdizadeh, Mehdizadeh & Baghaeifar, 2020; Meng et al., 2018; Sobiech et al., 2020). Therefore, some tank-mix adjuvants are able to reduce the amount of pesticide usage, enhance control efficacy, facilitate the delivery of pesticide chemicals, and alleviate environmental pollution (Jibrin et al., 2021; Li et al., 2021; Meng et al., 2018; Preftakes et al., 2019; Wang et al., 2018).

Although the effects of tank-mix adjuvants and UAV spraying on droplet distribution have been studied in a wide scope of crops and study objectives (Chen et al., 2021; Kim et al., 2019), very little research work has been carried out to evaluate the impacts of the combination of tank-mix adjuvant and UAV spraying on the citrus crop. In this paper, CAs of water-only sprays and adjuvant-presence sprays with five concentration levels are measured to evaluate the outperformance concentration for droplet distribution in citrus tree canopy. Two multi-rotor UAVs (DJI T20 and T30) which consist of different sizes of pesticide tanks and rotor diameters are adopted and the tank-mix adjuvant with optimization concentration is used to study the effects of the adjuvant on droplet distribution in the citrus tree canopy, by evaluating droplet coverage and droplet size in different canopy layers and droplet penetration in the canopy. This paper introduces the experiments undertaken, related results, analysis, and implications.

## Materials and Methods

### Field trial site

**Figure 1:**
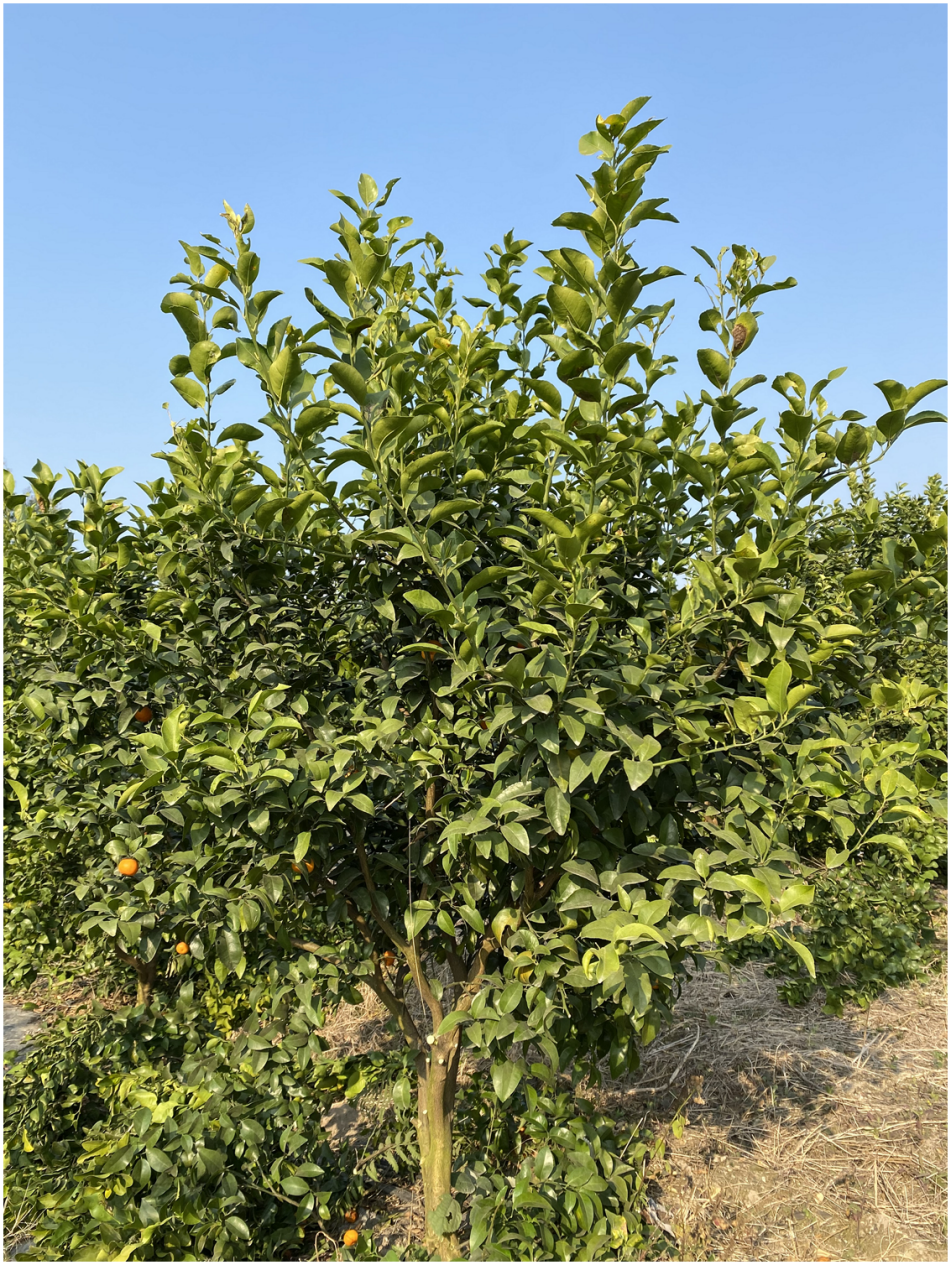
The target citrus tree Shatangju used in this work.

The field experiments were conducted at the Citrus Planting Base in Phoenix Overseas Chinese Farm, Xingbin District, Laibin City, Guangxi Zhuang Autonomous Region (E109°26′, N23°98′) and the experiments were done on January 24, 2021. Xingbin District is in the transition zone from south subtropical to mid-subtropical. It has strong solar radiation, sufficient sunshine, a warm climate, abundant rainfall, and long frost-free period, which is suitable for citrus cultivation. The annual average sunshine hours, average temperature, and average annual rainfall are 1,568.2 h, 20.7 °C, and 1,352.9 mm, respectively. The target citrus is Shatangju, which is widely cultivated in southern China. The tested citrus tree is 5 years old, with a crown width of 2∼2.5 m and an average plant height of 3 m. The interval between plants is 2 m and the row spacing is 4 m. The target citrus Shatangju is shown in Fig. 1.

### Spraying platform

**Figure 2:**
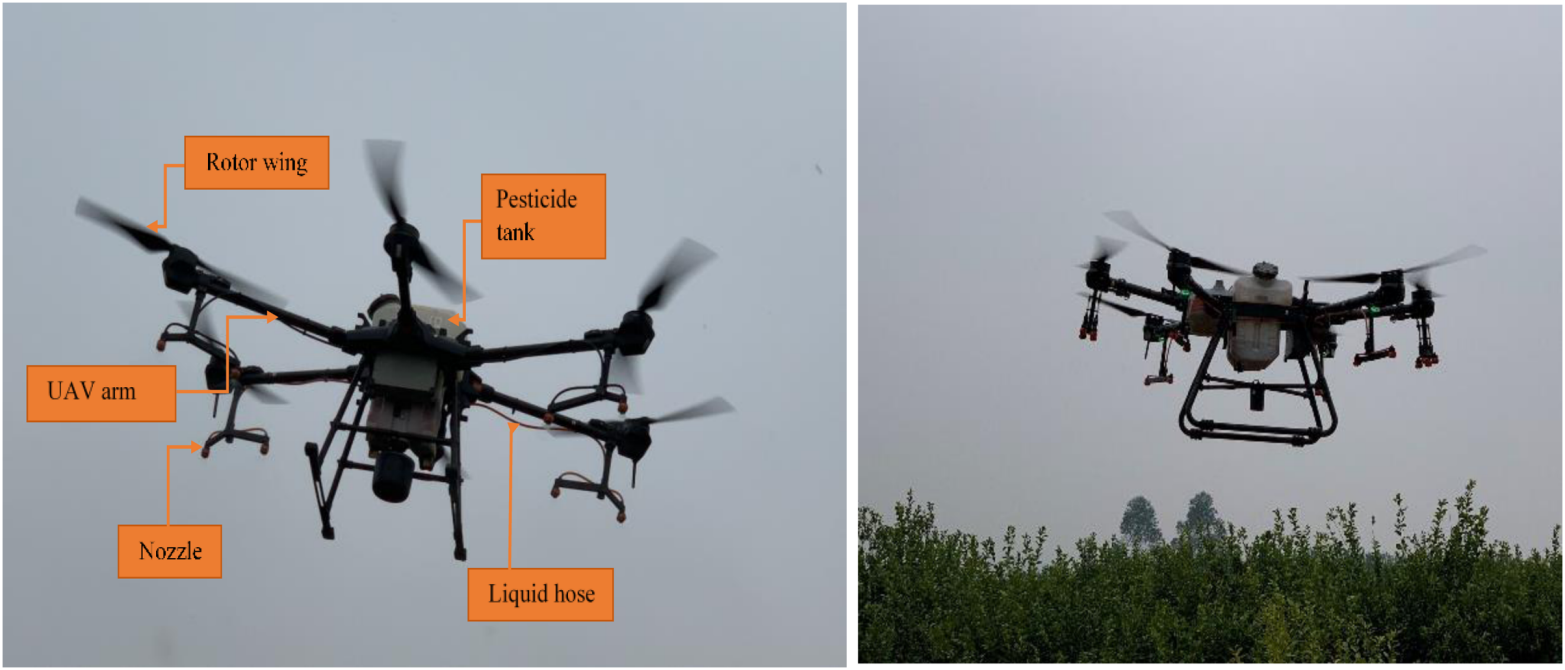
Two plant protection UAVs used in this study: T20 (left) and T30 (right).

The crop protection UAV models used in this experiment are DJI T20 and T30 regular version (Shenzhen DJI Innovation Technology Co., Ltd., Shenzhen, China). As shown in Fig. 2, when the arms and the blades of the two UAVs are deployed, the overall dimensions (length × width × height) of the two UAVs are 2,509 mm × 2,213 mm × 732 mm (T20) and 2,858 mm × 2,685 mm × 790 mm (T30 ordinary version), respectively. Both UAV models are six-rotor and adopt Real-Time Kinematic (RTK) positioning. The capacity of the pesticide tanks of T20 and T30 is 20 L and 30 L, respectively. Each UAV is equipped with a set of TEEJET flat-fan hydraulic nozzles 110015VS (T20) and 11001VS (T30). The measured single nozzle flow rate range of these two UAVs before spraying is 0.25∼0.42 L min^−1^ (2.8 bar). T20 is equipped with eight nozzles, each two of which are installed vertically under the rotors, respectively. T30 has sixteen nozzles, only twelve of which are opened during the spraying operation. In this field test, the operating height of the two UAVs is 2 m above the top canopy of the citrus trees. The flight velocity and application rate of both UAVs is 3 m s^−1^ and 75 L ha^−1^, respectively.

### Tank-mix adjuvant

The brand name of a typical tank-mix adjuvant used in this study is Nong Jian Fei (NJF), which is produced and provided by Guilin Jiqi Biochemical Co., Ltd. NJF belongs to organosilicon-based spray auxiliaries, which has been widely used in pesticide application process (Meng et al., 2021).

### Experimental design

The experiment is divided into two parts, the first part is for dynamic CA measurement on the adaxial surface of citrus leaves in the indoor lab and the second part is for droplet distribution assessment in the citrus tree canopy on orchard farm.

### Dynamic CA measurement

**Figure 3:**
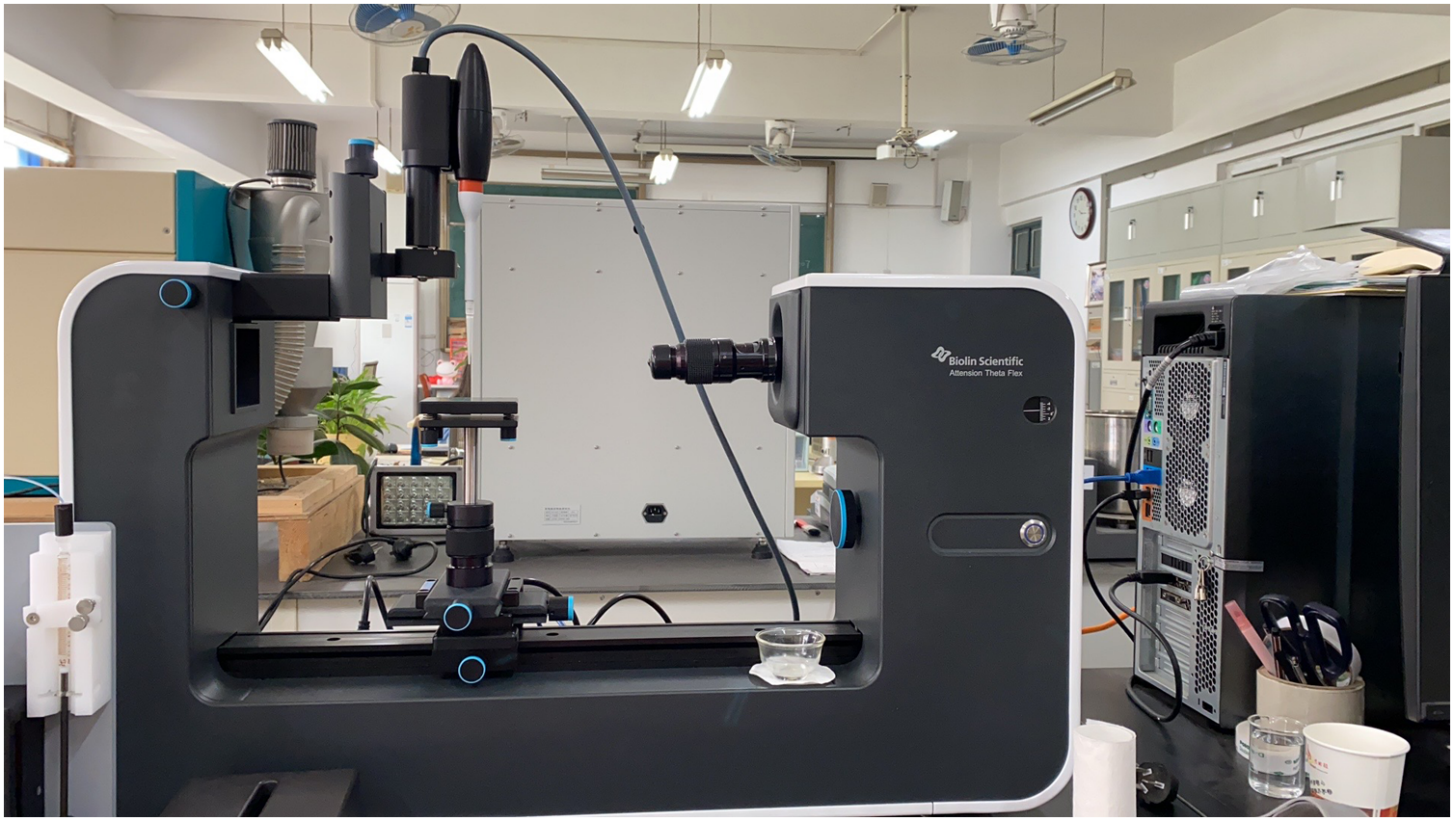
The optical tensiometer for measuring CA in this study.

**Figure 4:**
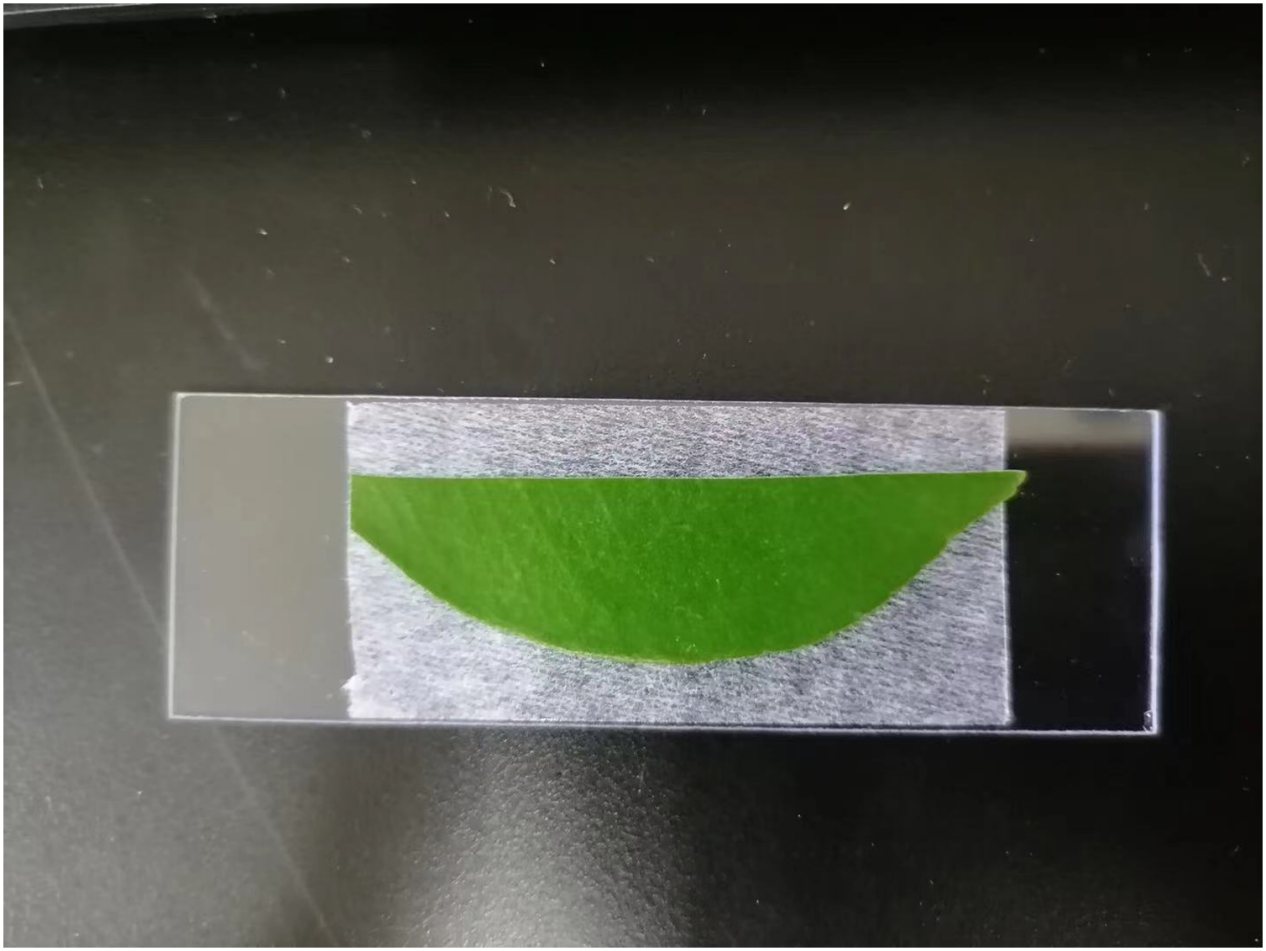
The citrus leaf for measuring CA on a glass slide.

The sessile drop method is usually employed to check the wettability of the solid surface due to its simplicity and quick measurement. In this study, the sessile drop method is utilized for measuring CA to determine the spread ability of NJF-presence sprays on the adaxial surface of citrus leaves. In this method, the optical tensiometer Attention Theta Flex (Biolin Scientific, Beijing, China), equipped with a high-resolution camera (1,984 × 1,264 px with a maximum of 3009 FPS) and LED light, is adopted to measure CA (Fig. 3). The droplet will sit on the surface of the sample leaf after it leaves the disposable tip dispenser. When the three-phase boundary of the sitting droplet is moving, dynamic CA can be measured by the instrument. In this study, the dynamic CA of water-only sprays and NJF-presence sprays are measured, respectively. Five concentrations (0.05‰, 0.1‰, 0.2‰, 0.3‰, and 0.5‰) of NJF-presence aqueous solution are used in the experiments and three replications are performed for each concentration. The tip size of the dispenser is 300 μL and the droplet release rate is set at 4 μL s^−1^ for all treatments. All tested leaves are sectioned along the leaf vein and then placed onto a glass slide of 25 cm × 76 cm to reduce undulation and facilitate the capture of images for CA measurement (Fig. 4). The images are captured at 140 FPS during 10 s and the dynamic CA is measured from 0.00 to 10.00 s. The dynamic CA is analyzed by every image captured. Laboratory experiments are performed at a constant relative humidity of 55% and room temperature of 25 ± 0.2 °C.

### Droplet distribution measurement

**Figure 5:**
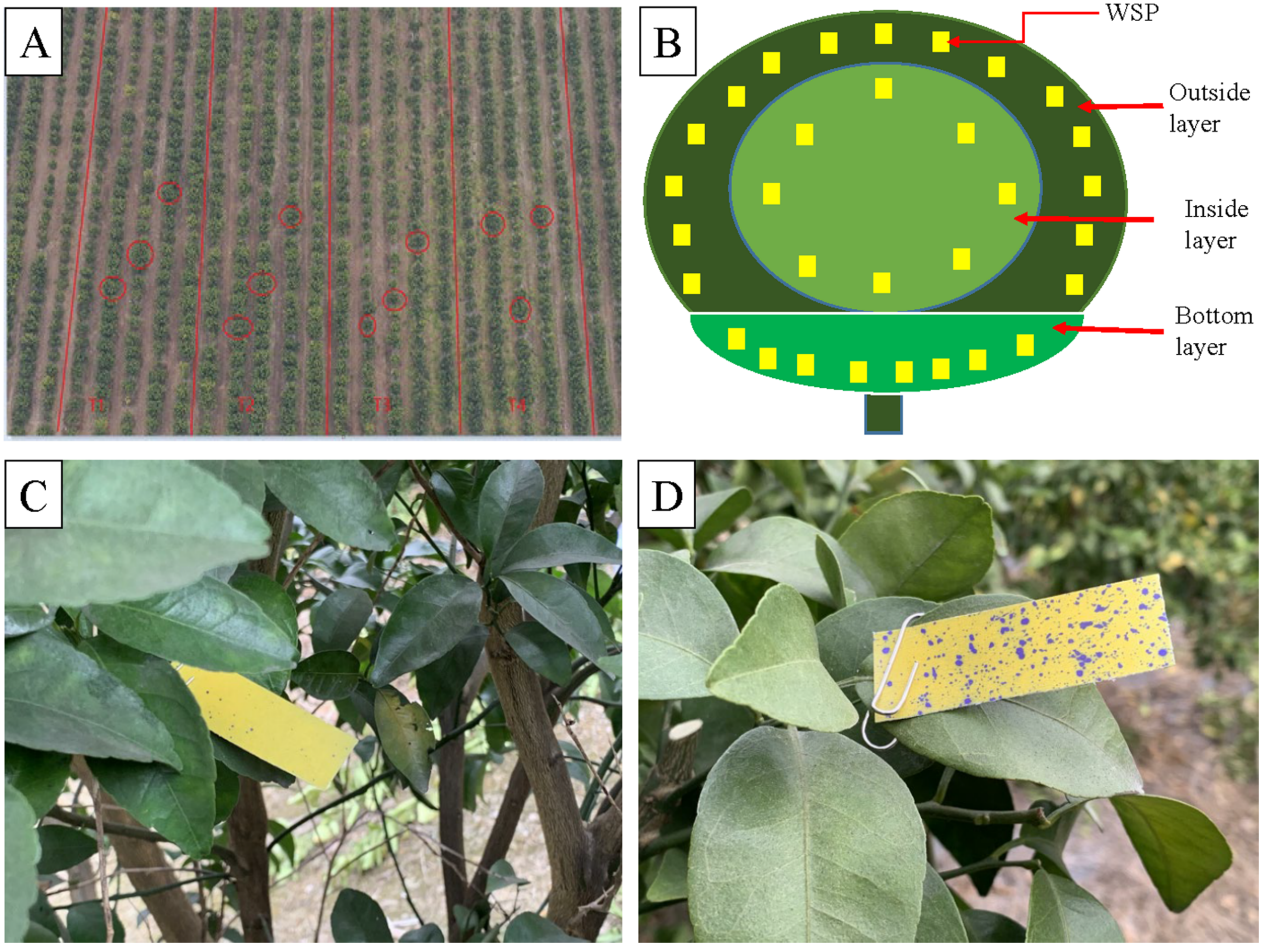
Treatment arrangements (A), sampling layers of citrus tree canopy (B), sprayed WSP in inside layer (C) and outside layer (D).

According to the geometric traits of the canopy shape of the Shatangju trees, both UAVs are operated above the trees and fly along the planting row, respectively. The NJF-presence sprays with a concentration of 0.5‰ is prepared according to the results of CA measurement before the field spraying test. The arrangement of four treatments, sampling layers, and WSP placement are shown in Fig. 5. Four treatments are designed for measuring droplet distribution and three trees are selected randomly for repetition within the treatment (Table 1 and Fig. 5A). Water Sensitive Papers (WSPs) are placed to collect droplets according to the sketch map of WSP placement (Fig. 5B). Both water-only and NJF-presence treatments are conducted for both UAVs, respectively. The size of each treatment area is 20 m × 100 m. According to the shape characteristics of the target trees and the vertical and horizontal directions, the tree canopy is divided into three layers, outside, inside, and bottom, as illustrated in Fig. 5B. The bottom layer starts from the lowest leaf of the canopy and extends upward around 30 cm. Except for the bottom layer, the outside layer is located from the edge of the canopy to about 30 cm inside, while the further inside part is referred to as the inside layer. Starting from the leftmost end of the UAV’s forward direction, fifteen labeled WSPs are evenly arranged on the outside layer of the canopy in a clockwise direction. Eight labeled WSPs are also evenly arranged in a clockwise direction in the inside and bottom layers, respectively.

**Table 1:** Treatments of the flied experiment.

Treatments	UAV	Adjuvant NJF
T1	T20	×
T2	T20	√
T3	T30	×
T4	T30	√

**Note:**

“√” denotes aqueous solution with NJF while “×” indicates water-only sprays.

### Meteorological conditions

Temperature and relative humidity are measured using the Kestrel 5500 Link NK (Kestrel Company, Louisville, KY, USA) handheld comprehensive weather station. The field trial day is cloudy, and the average temperature and relative humidity is 21 ± 2.1 °C and 77 ± 2.4%, respectively.

### Data processing and analysis

WSPs are scanned and then processed in an image-based droplet analysis system DepositScan (USDA, Washington, DC, USA). Droplet coverage and Volume Median Diameter (VMD) are derived by the analysis system. Origin 2019 (Academic) (Origin Lab, Northampton, MA, USA) is used to draw the figures and analyze the differences at a significance level of 0.05 between treatments using Tukey’s method in one-way ANOVA.

## Results

### CAs of water-only and aqueous solution of NJF

**Figure 6:**
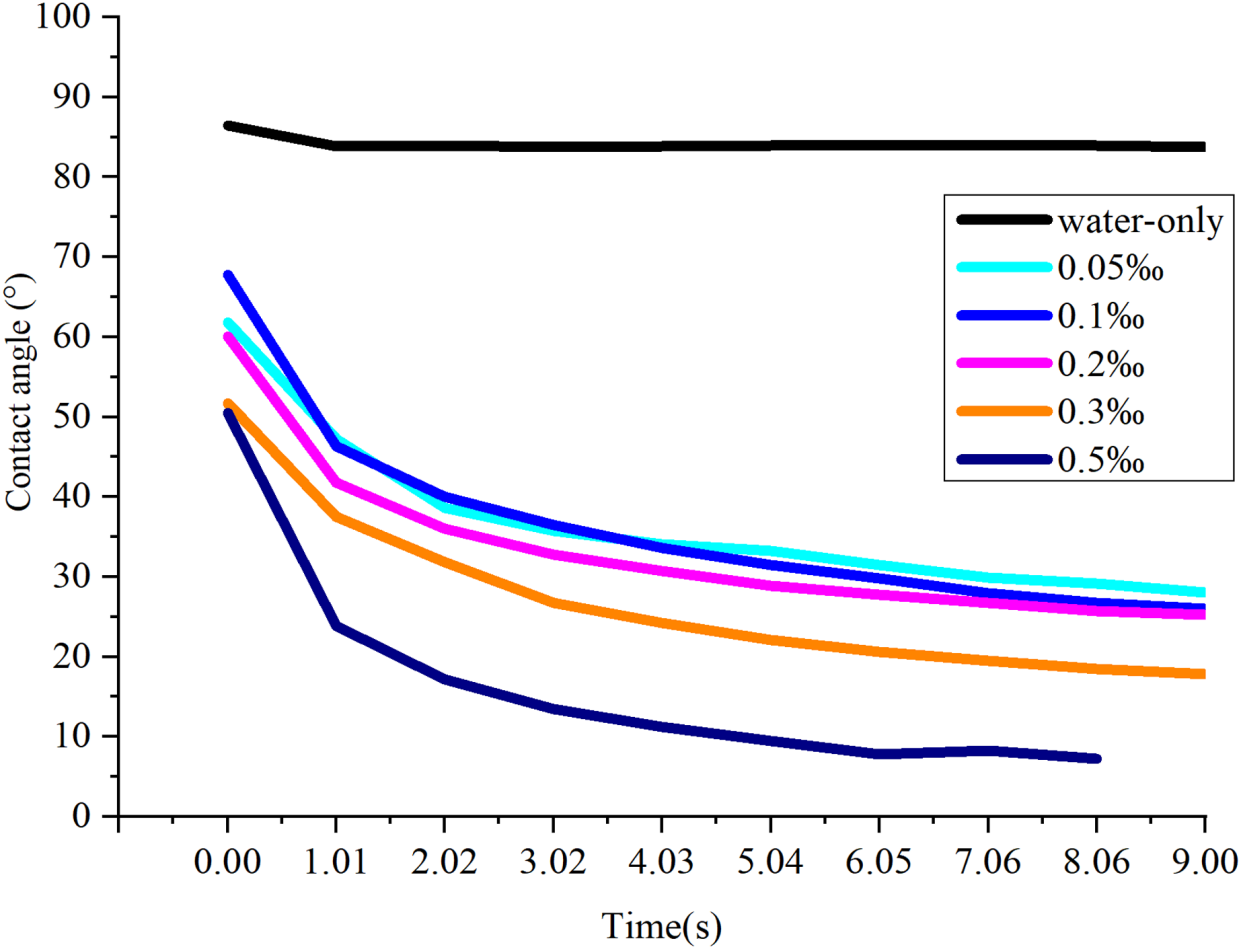
The effect of NJF concentrations on CA reduction.

**Figure 7:**
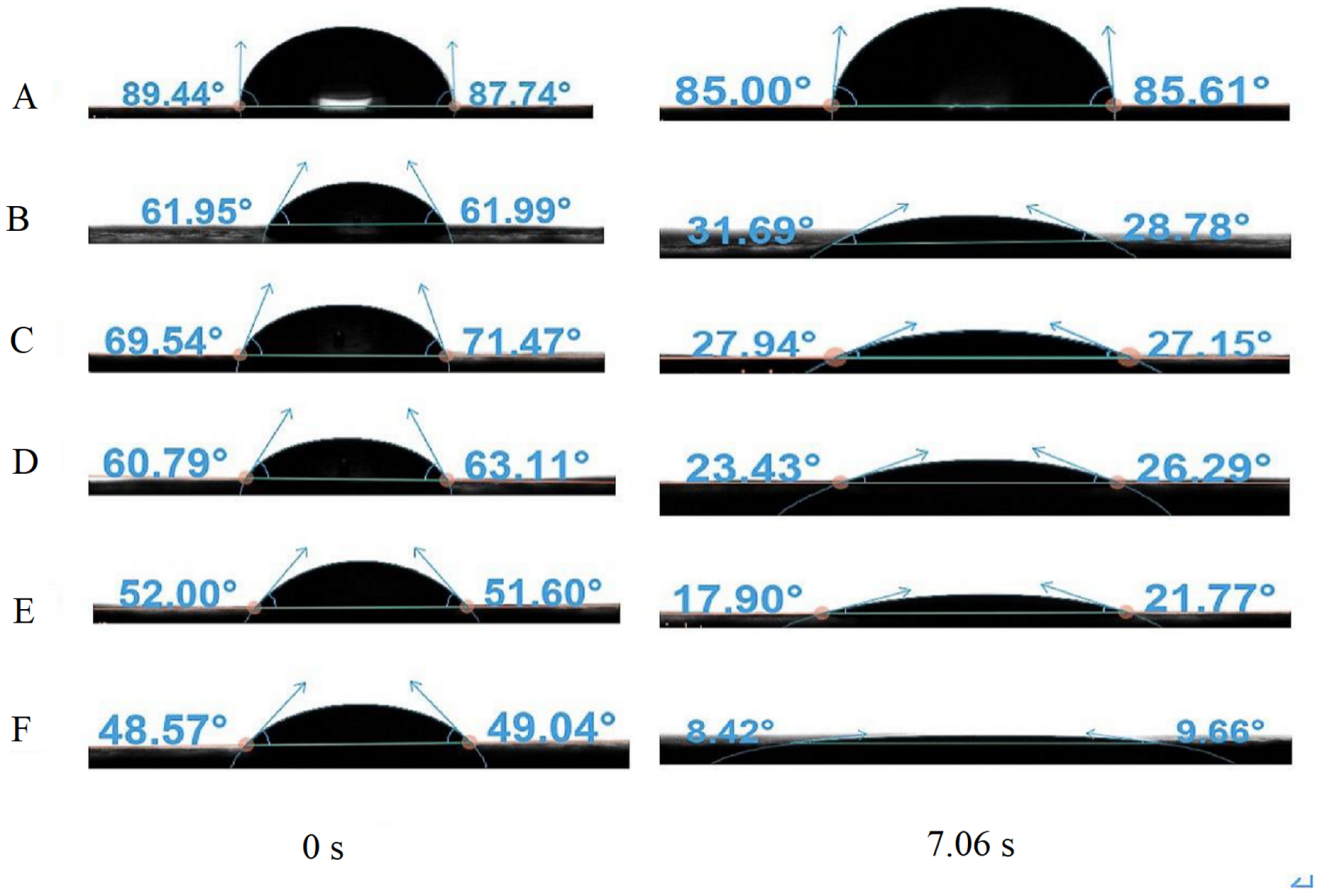
The CAs of aqueous solution containing NJF with different concentrations at 0.00 s and 7.06 s. (A) water-only; (B) 0.05‰; (C) 0.1‰; (D) 0.2‰; (E) 0.3‰; (F) 0.5‰.

As shown in Fig. 6, the CA of water-only sprays is substantially higher than that of the NJF-presence sprays during all observing time. It stays stable around 83.8° during the measuring time except the moment droplet touches the sample leaf (0.00 s, 86.4°). The addition of NJF significantly reduces the CAs of the sprays at 0.00 s. Furthermore, the CAs of these sprays decrease rapidly after 0.00 s and reduce by 14.0∼27.0° at 1.01 s. For NJF-presence sprays with concentrations of 0.05‰, 0.1‰, and 0.2‰, there is little difference in CA attenuation. At 9.00 s, the CAs of the sprays with these three concentrations are 25.2∼28.0°, which account for around 38∼45% of the CAs at 0.00 s. For the sprays with concentrations of 0.3‰ and 0.5‰, the CAs at 0.00 s are almost the same (50.5∼51.7°), and the trend of CA reduction of these two concentrations is similar to the previous three concentrations. However, at 1.01 s, the CAs of the sprays with a concentration of 0.3‰ and 0.5‰ drop rapidly to 37.5° and 23.9°, respectively. The CAs of these two concentrations are further reduced to below 20.0° at 7.06 s (Fig. 7). It can be observed from Figs. 6 and 7 that the NJF addition makes CA reduce gradually as the observing time goes on. Furthermore, at a specific observation time point, the higher the concentration is, the more reduction of CA is.

### Droplet coverage

**Figure 8:**
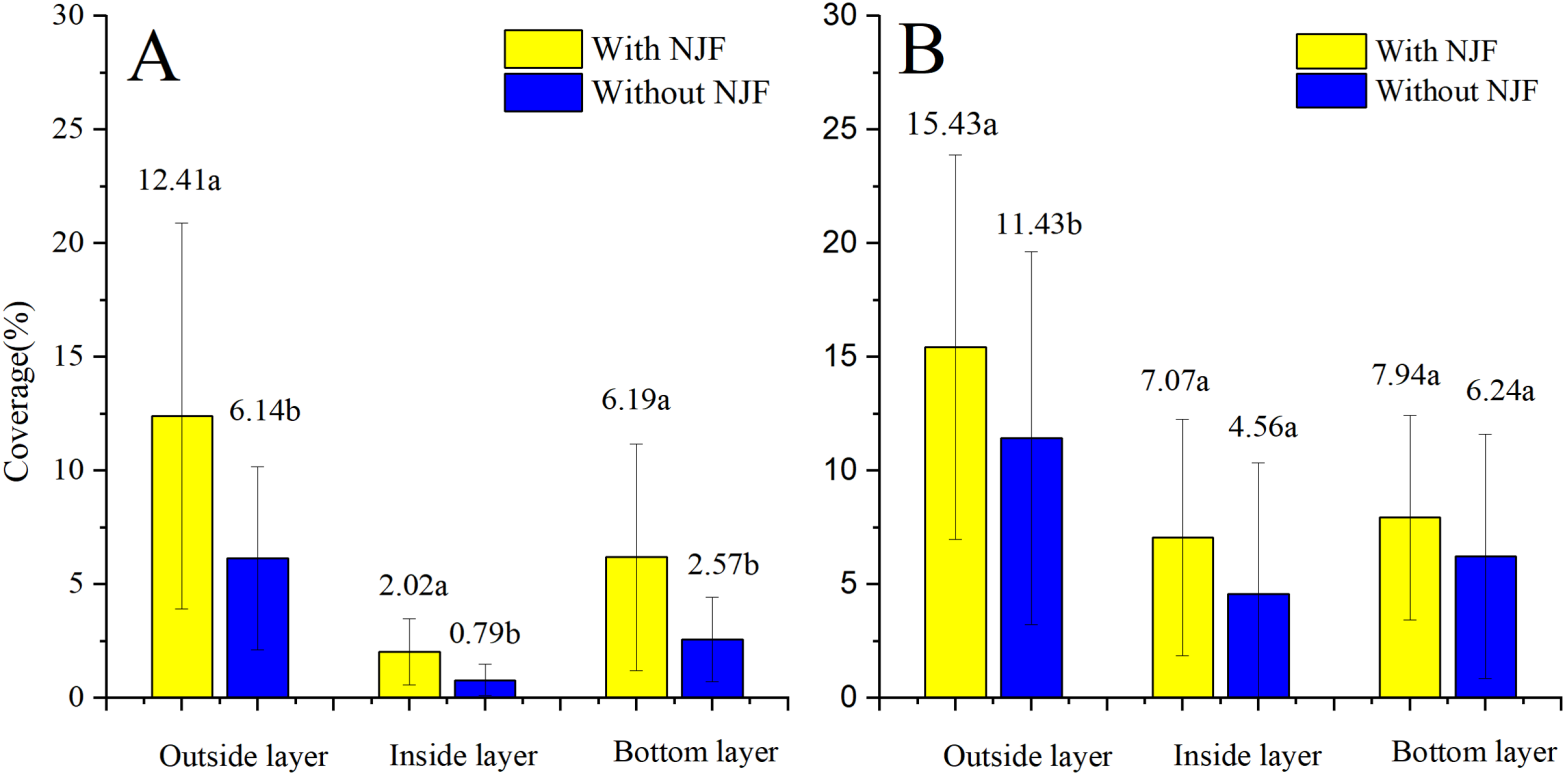
Droplet coverages of T20 (A) and T30 (B) with and without NJF in outside, inside, and bottom layers. Different lowercase letters indicate significant difference in the same layer at 0.05 level by Turkey test.

The comparison of droplet distribution for the same UAV before and after NJF addition is illustrated in Fig. 8. Droplet coverages in three layers of the citrus canopy are significantly increased by the addition of NJF when T20 is adopted as the sprayer. However, when T30 is used as the spraying platform, NJF addition only notably influences droplet coverage of the outside layer. In the inside and bottom layers, the difference between treatments with and without NJF is much smaller than that of the outside layer, which is shown to be with no statistically significant difference. For T20, droplet coverage of the treatment with NJF (12.41%) is around two times of the treatment without NJF (6.14%) in the outside layer. In the inside and bottom layers, droplet coverage of the treatment with NJF is about 2∼3 times of the treatment without NJF. For T30, outside layer droplet coverage of the treatment with NJF (15.43%) is around 1.35 times of the treatment without NJF. In the bottom layer, droplet coverage values of the treatment with and without NJF are very close. It can be also derived from Fig. 8 that the outside layer obtains 51∼65% of the collected droplet coverage for both UAVs before adding NJF into the sprays, while that of the treatments with NJF is 51∼60%. The reduction of droplet coverage percentage indicates that droplet penetration might be improved by NJF addition for some UAV spraying. For T20, the percentages of droplet coverage in the inside and bottom layers are increased from 8% to 10% and 27% to 30%, respectively. In the outside layer, the percentage is decreased from 65% to 60%. When T30 is used as the spraying platform, the percentage of droplet coverage in the inside layer increases from 21% to 23%, while that of the bottom layer decreases from 28% to 26% and that of the outside layer stays the same (51%).

**Figure 9:**
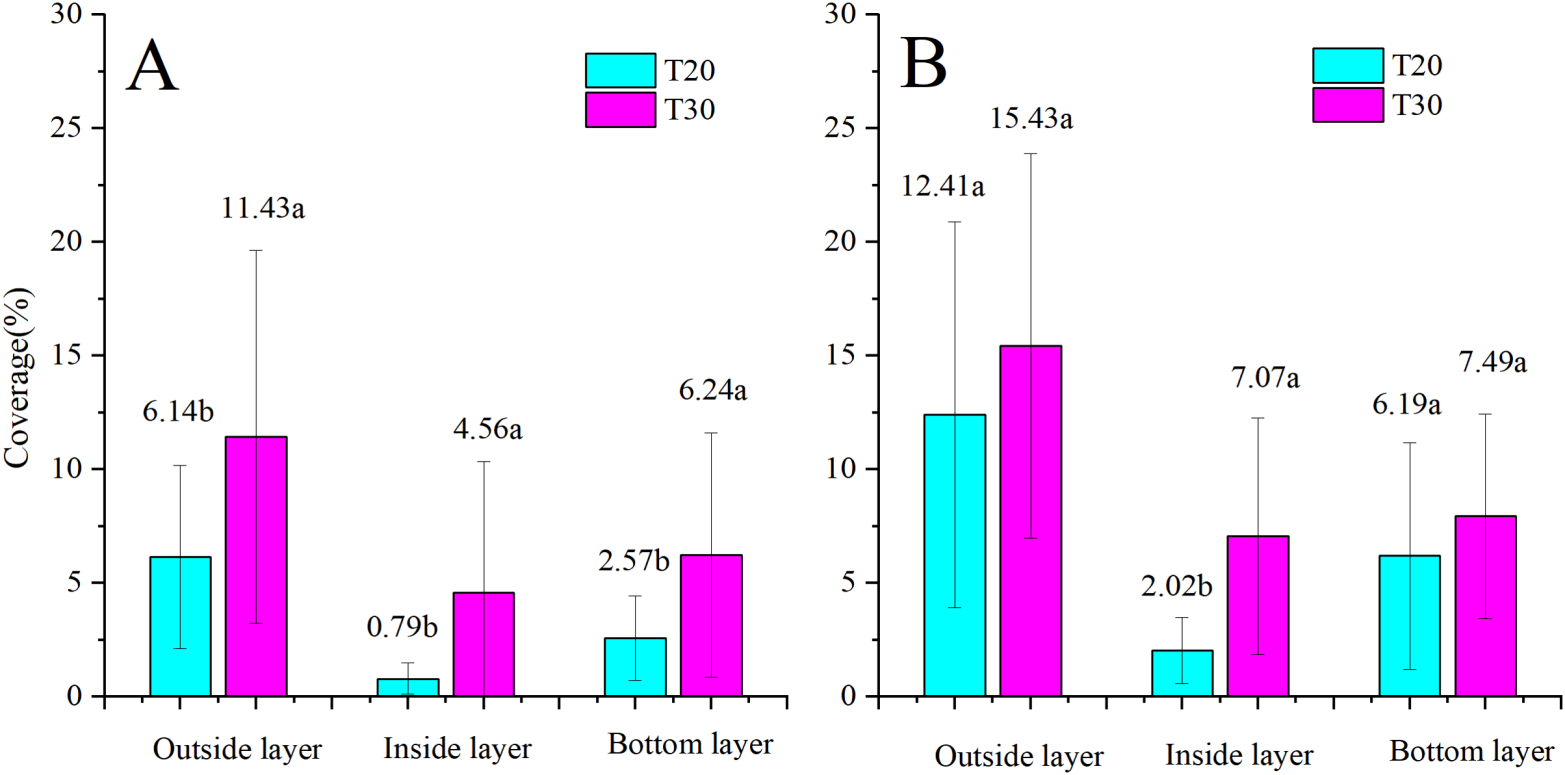
Droplet coverages of treatments without (A) and with (B) NJF sprayed by T20 and T30 in outside, inside, and bottom layers. Different lowercase letters indicate significant difference in the same layer at 0.05 level by Turkey test.

The comparison of the droplet coverage changes between T20 and T30 before and after the NJF addition is shown in Fig. 9. Before NJF addition, droplet coverage of T30 is significantly higher than that of T20 in three canopy layers, respectively. However, with the addition of NJF, no significant difference is observed for the droplet coverage of the outside and bottom layers between T20 and T30, respectively. The notable difference is observed between T20 and T30 in the inside layer after the addition of NJF. Furthermore, the average droplet coverage of treatment with NJF in the whole canopy is around 1.35 times (T30) and 2.15 times (T20) of the treatment without NJF, correspondingly. These results imply that the addition of a tank-mix adjuvant might shorten the differences of droplet coverages among different UAVs.

### Droplet size (VMD)

**Figure 10:**
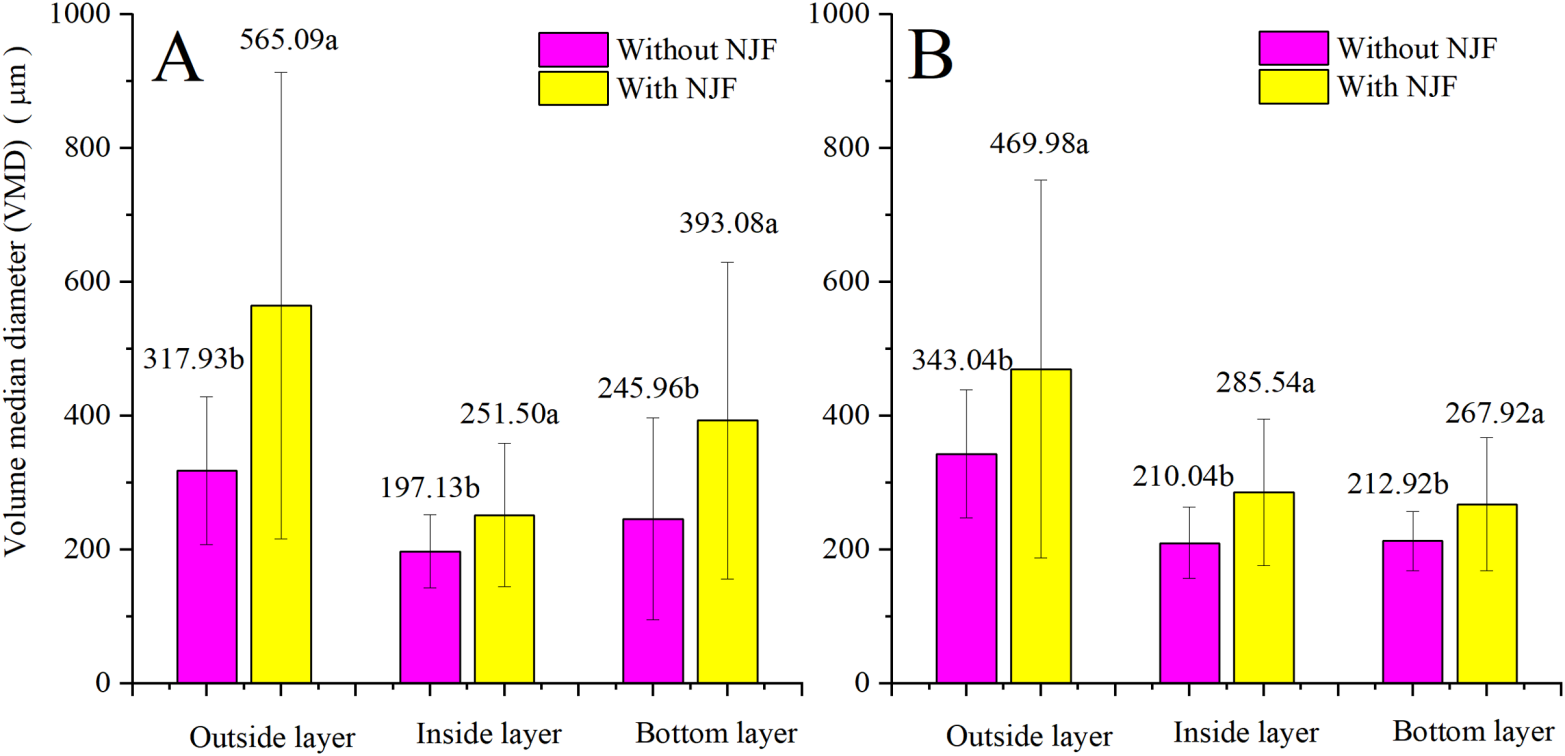
VMDs of T20 (A) and T30 (B) with and without NJF in outside, inside, and bottom layers. Different lowercase letters indicate significant difference in the same layer at 0.05 level by Turkey test.

The comparison of the effects of NJF addition on VMD between treatments of the same UAV is illustrated in Fig. 10. The addition of NJF has a notable effect on the VMD of three layers for both UAVs. The VMDs of sprays with NJF are significantly increased compared to those of the water-only sprays. For T20, the VMDs of the droplets in the outside layer are increased by around 78% due to NJF addition compared to these of the treatments without NJF. However, the corresponding increment of T30 is only around 37%. The largest VMD is observed in the outside layer for both UAVs, regardless of with and without NJF in the spraying liquids. For T20, the second largest and the smallest droplet size are found in the bottom layer and inside layers for both treatments with and without NJF. For T30, droplet sizes in the inside and bottom layers are quite close for both treatments with and without NJF. It is indicated that the opportunities of the coarse droplets depositing on leaves in the inside and the bottom layer is almost equal when T30 is adopted.

**Figure 11:**
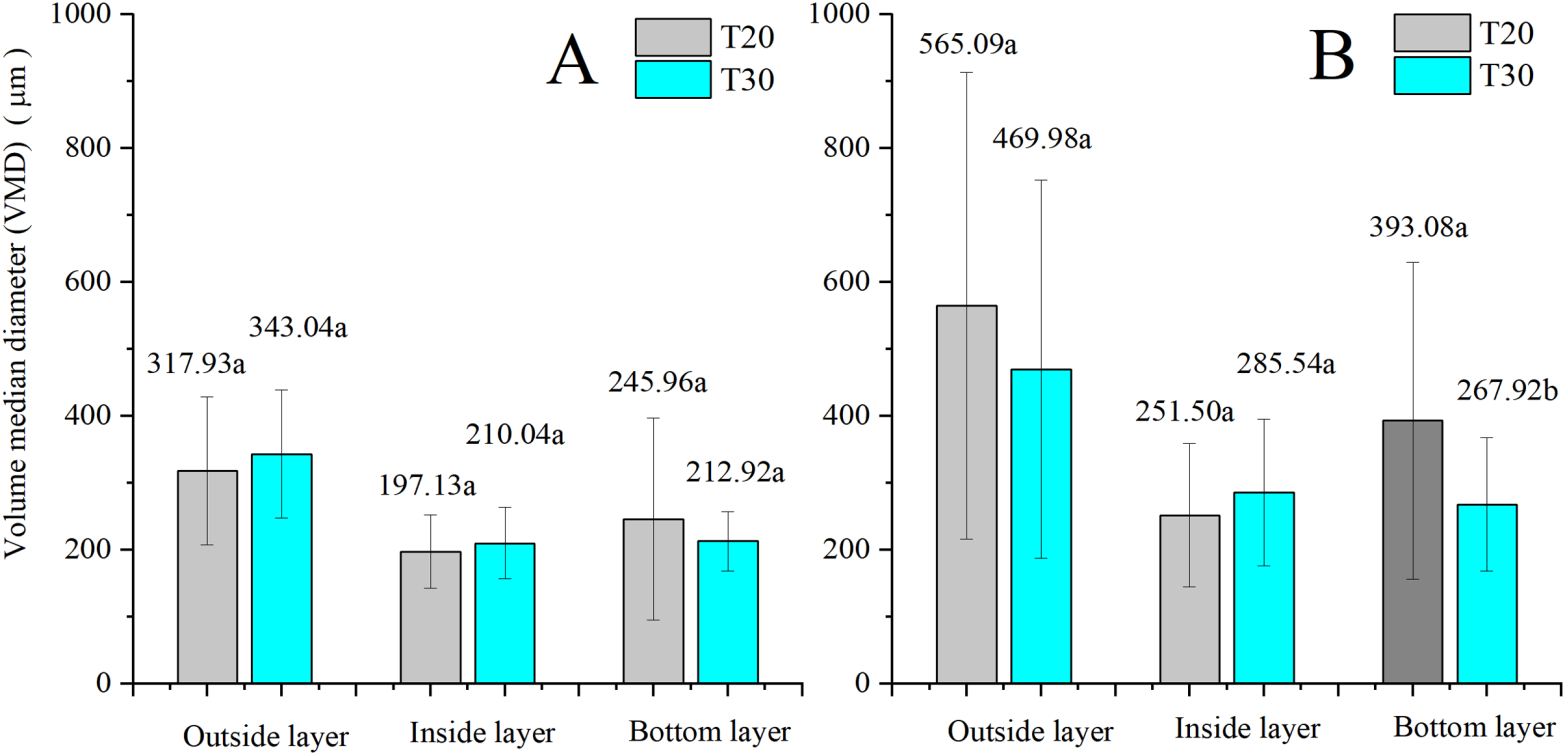
VMDs of treatments without (A) and with (B) NJF addition by T20 and T30 in outside, inside, and bottom layers. Different lowercase letters indicate significant difference in the same layer at 0.05 level by Turkey test.

The comparison of the effects of NJF addition on droplet size between two UAVs is shown in Fig. 11. For water-only sprays, the descending order of VMD of both UAVs can be observed to be outside, bottom, and inside, and no significant difference is observed between the VMDs of T20 and T30 in three layers, respectively. After the addition of NJF, the VMDs of both UAVs in three layers are increased. However, the VMDs of two UAVs in the outside and the inside layers have no remarkable difference, while that of the bottom layer shows the significant difference between T20 and T30.

### Correlation coefficient of droplet coverage and droplet size

During the application of pesticides, maximizing the droplet coverage and adopting appropriate droplet size are required to get a satisfactory pesticide effectiveness (Ferguson et al., 2016, 2020). To further analyze the effects of NJF addition on droplet coverage and droplet size, the correlation coefficient (CC) between these two observed metrics is calculated using the following Eq. (1).

(1) $$\matrix{ {CC\left({dc,vmd} \right) = \displaystyle{{\sum \left( {dc - \overline {dc} } \right)\left( {vmd - \overline {vmd} } \right)} \over {\sqrt {\sum {{\left( {dc - \overline {dc} } \right)}^2}\sum {{\left( {vmd - \overline {vmd} } \right)}^2}}}}} \cr }$$

Where, *dc* represents droplet coverage and *vmd* indicates Volume Median Diameter (VMD).

As shown in Table 2, all CCs of T20 in three layers are increased after the addition of NJF, of which, the CC of the outside layer is the largest. The CC of water-only sprays is 0.45, while that of the sprays with NJF reaches 0.93. For T30, the CCs of the bottom and outside layer increase with the addition of NJF, while that of the inside layer decrease slightly after NJF addition. The increase of CC indicates that both droplet coverage and VMD increase at the same time after the NJF addition.

**Table 2:** Correlation between droplet coverage and droplet size.

Treatments	Canopy layer	CC of droplet coverage and VMD
T20 without NJF	Bottom	0.32
T20 with NJF	Bottom	0.81
T20 without NJF	Inside	0.43
T20 with NJF	Inside	0.65
T20 without NJF	Outside	0.45
T20 with NJF	Outside	0.93
T30 without NJF	Bottom	0.50
T30 with NJF	Bottom	0.59
T30 without NJF	Inside	0.67
T30 with NJF	Inside	0.65
T30 without NJF	Outside	0.77
T30 with NJF	Outside	0.93

## Disussion

From the above results of this study, it can be summarized that the addition of a typical tank-mix adjuvant NJF has significant effects on CA reduction, droplet size and droplet coverage increase, and penetration improvement for a certain type of UAV. Furthermore, the high enough concentration of NJF is of great importance in achieving the great reduction of CA. The highest concentration (0.5‰) contributes to the greatest and the most rapid reduction of CA during the observing time, which is confirmed by the results of CA measurement. Thus, NJF with a concentration of 0.5‰ is adopted in the field trial for evaluating droplet distribution in the citrus canopy. Some of the organosilicon-based adjuvants have greater effects on reducing the CA of aqueous solutions than conventional adjuvants (Meng et al., 2021; Singh & Mack, 2010; Stevens, 1993). In this study, NJF with appropriate concentrations has a great ability to reduce CA, which is consistent with the previous research. A large CA can be observed when the liquid beads on the solid liquid, while a small CA can be observed when the liquid spreads on the solid liquid (Yuan & Lee, 2013). In this study, the CA reduction of the spray liquids might facilitate droplets spreading on the WSPs, which might contribute to the increase of droplet size and coverage. In addition to the impact of adjuvant, size of nozzle orifice also affects droplet size (Ferguson et al., 2016). UAVs equipped different nozzles might have different droplet size, which might influence droplet distribution. However, although the two UAVs are equipped different sizes of nozzles, no significant difference is investigated between the VMDs of the two UAVs in the same canopy layer before NJF addition. Furthermore, there is also no significant difference between the VMDs of the two UAVs in the outside and inside layer after NJF addition. Since WSP is hydrophilic, the influence of droplet expansion on WSPs might be the reason for this phenomenon. With regards to the individual UAV, after NJF addition, the VMDs of the two UAVs are remarkable larger than that of the water-only sprays in the same canopy layer, respectively. This further indicates that NJF addition could increase droplet size. The change of droplet size further influences droplet distribution and penetration in the canopy (Chen et al., 2020). The small droplets tend to deposit in the bottom and inside layers of the canopy (Chen et al., 2020; Ferguson et al., 2016), this also is verified by the results of droplet size measurement in this work.

Large droplet size can improve the droplet coverage in the upper and lower crop canopy (Chen et al., 2020). From the results of droplet coverage measurement, it can be observed that the addition of NJF has the effects on increasing penetration of droplets in citrus canopy by T20. The percentages of droplet coverage in the lower canopy (bottom and inside layers) are increased after the addition of NJF. However, when T30 is used as the spraying platform, the addition of NJF has a slight effect on droplet penetration. The percentages of droplet coverage in the outside layer (51%) is not affected by NJF addition, while that of the inside layer increases slightly from 21% to 23% and that of the bottom layer decreases slightly from 28% to 26%. The droplet penetration of T30 is better that that of T20, regardless with or without NJF addition. The results might indicate that the effect of NJF addition on droplet penetration is associated with the UAV structure dimension. UAV structure dimension has an impact on the downwash airflow generated by UAV rotors. Downwash distribution has a high correlation with droplet distribution (Guo et al., 2019; Guo et al., 2021; Wu et al., 2019). The previous study shows that the distribution of downwash is significantly affected by the size of the rotor and dimensions of the UAV (Conlisk, 1997; Tang et al., 2017). The size of T30 (2,858 mm × 2,685 mm × 790 mm) is bigger than that of T20 (2,509 mm × 2,213 mm × 732 mm), which might generate stronger downwash. The effects of strong downwash might be more significant than that of the NJF addition.

All CCs of droplet coverage and droplet size (VMD) are increased with the addition of NJF except that of the inside layer of T30. As indicated by the results, no significant difference is observed between droplet coverage and VMD of T30 in the inside layer due to the addition of NJF, which might be the reason for the decrease of CC of the inside layer of T30. These results further explain that NJF addition is more pronounced to enlarge both droplet coverage and droplet size in most cases. As the key spraying parameters of UAV spraying, a larger droplet coverage might have more chances to get a better control efficacy and a larger droplet size is more suitable for drift reduction in off-target area (OECD, 2021; Wang et al., 2018). However, more data points are required in future work to draw a more solid conclusion on this finding along with in-depth explanation.

## Conclusions

The conclusions are drawn as follows according to the results and the discussions:

1. The typical tank-mix adjuvant NJF has a significant effect on reducing the CA of the aqueous sprays. A high enough concentration (*e.g*., 0.5‰ NJF aqueous solution) is required to get a satisfactory CA reduction for a better droplet spread on the targets.
2. For the same UAV, the addition of the adjuvant significantly increases both the droplet coverage and droplet size, which might contribute to the increase of CCs between these two parameters.
3. The difference of droplet coverages among the UAVs could be mitigated by the addition of the adjuvant.
4. Droplet penetration of a certain type of UAV (*e.g*., T20) can be improved by the addition of the adjuvant.

Therefore, the addition of a tank-mix adjuvant with the ability to reduce CA of the aqueous sprays, can effectively improve droplet distribution using UAV spraying in the citrus canopy by increasing droplet coverage and VMD.

## Supplemental Information

10.7717/peerj.13064/supp-1 Supplemental Information 1 Contact angles of water-only and sprays with NJF adjuvant.

10.7717/peerj.13064/supp-2 Supplemental Information 2 Droplets of T30.

10.7717/peerj.13064/supp-3 Supplemental Information 3 Droplets of T20.

## Additional Information and Declarations

### Data Availability

The following information was supplied regarding data availability: The raw measurements are available in the Supplemental Files.
